# Efficacy and Safety of Qinpi Tongfeng Formula in the Treatment of Acute Gouty Arthritis: A Double-Blind, Double-Dummy, Multicenter, Randomized Controlled Trial

**DOI:** 10.1155/2022/7873426

**Published:** 2022-07-12

**Authors:** Yihua Fan, Wei Liu, Hang Lu, Jian Liu, Rui Wu, Jun Zhao, Aihua Wang, Xianheng Zhang

**Affiliations:** ^1^First Teaching Hospital of Tianjin University of Traditional Chinese Medicine, Tianjin 300193, China; ^2^National Clinical Research Center for Chinese Medicine Acupuncture and Moxibustion, Tianjin 300381, China; ^3^Hospital of Chengdu University of Traditional Chinese Medicine, Chengdu 610075, Sichuan, China; ^4^The First Affiliated Hospital of Anhui University of Chinese Medicine, Hefei 230031, Anhui, China; ^5^The First Affiliated Hospital of Nanchang University, Nanchang 330006, Jiangxi, China

## Abstract

**Objective:**

Traditional Chinese medicine (TCM) has certain curative effect against acute gouty arthritis (AGA), but it lacks high-quality evidence-based studies. In this randomized controlled trial, we try to evaluate the clinical efficacy and safety of Qinpi Tongfeng Formula (QPTFF) in the treatment of AGA.

**Methods:**

One hundred and fourteen patients with AGA (damp heat accumulation syndrome) who met the inclusion and exclusion criteria were randomly divided into treatment group and control group in a ratio of 1 : 1. Patients in the treatment group were treated with QPTFF, and patients in the control group were treated with diclofenac sodium sustained-release tablets for 7 days. The primary outcome measure was the change in visual analog scale (VAS) score for pain from the baseline to day 8. The secondary outcome measures were joint symptom score, TCM syndrome score, total effective rate, pain cure rate, complete pain relief time, patient satisfaction score, erythrocyte sedimentation rate (ESR), C-reactive protein (CRP), and serum uric acid level. The safety outcome measures were routine blood test, urinalysis, liver function including alanine aminotransferase and aspartate aminotransferase, renal function including blood urea nitrogen and serum creatinine, and the rate of treatment-related adverse events (TRAEs).

**Results:**

105 patients with 53 in the treatment group and 52 in the control group completed the 7-day treatment. There was no significant difference between two groups in demographic characteristics, VAS score for pain, joint symptom score, TCM syndrome score, ESR, CRP, and serum uric acid level before enrollment at baseline (based on both the full analysis set (FAS) and per protocol set (PPS), *P* > 0.05). The 95% confidence interval of the difference between the eighth and first VAS score for pain of the two groups was (−0.57, 0.42) in FAS and (−0.48, 0.47) in PPS. The lower bound of both FAS and PPS is greater than the bound value of −0.7. On day 8, there was no significant difference between the two groups in joint symptom score, TCM syndrome score, total effective rate, pain cure rate, complete pain relief time, patient satisfaction score, ESR, and CRP (FAS and PPS, *P* > 0.05). The serum uric acid level and TRAEs in the treatment group were significantly lower than those in the control group (FAS and PPS, *P* < 0.05).

**Conclusions:**

QPTFF could alleviate the symptoms of patients with AGA, which is not inferior to diclofenac sodium sustained-release tablets in analgesic. Moreover, QPTFF overmatches diclofenac sodium sustained-release tablets in decreasing serum uric acid level and TRAEs. Therefore, the results provide reliable foundation for QPTTF in the treatment of AGA. *Trial Registration.* This study protocol was registered in Chinese Clinical Trial Registry (registration number: ChiCTR2100050638).

## 1. Introduction

Gout is a metabolic rheumatism caused by abnormal purine metabolism, increased synthesis, and/or decreased excretion of uric acid, resulting in increased serum uric acid. With the high serum uric acid level, urate will be deposited into crystals to gather in joints, cartilage, and kidney [1]. Urate crystals in joints can lead to bone injury by repeated inflammation stimulation further to influence the daily activities of gout patients. Urate crystals in kidney can lead to gouty nephropathy which will develop into renal failure and endanger life, once poorly controlled [2]. In addition, long-term high serum uric acid level can increase the risk of cardiovascular events and cerebrovascular diseases [3].

Acute gouty arthritis (AGA) is a common acute arthritis with clinical features of severe arthralgia with swelling, recurrence, and poor prognosis [4]. For AGA, 2021 Asia Pacific League of Associations for Rheumatology [5] recommended anti-inflammatory and analgesic therapy. Colchicine and nonsteroidal anti-inflammatory drugs (NSAIDs) are the first-line drugs for the treatment of AGA. Although colchicine can alleviate the patient's condition in a short time, there will be different adverse reactions such as damage to the liver, kidney, gastrointestinal, and bone marrow suppression after the treatment [6]. NSAIDs can also effectively reduce joint pain, but they are lack in reducing serum uric acid level with certain side effects [7]. Hence, it is important to seek a safe and effective treatment method.

In traditional Chinese medicine (TCM), the clinical manifestations of AGA, such as joint swelling and tenderness and local skin redness, constitute the “damp heat accumulation syndrome” of “Bi syndrome” (joint pain) similar to the acute stage of gouty arthritis. The treatment of AGA with TCM has a history of thousand years in China. A preliminary systematic study [8]found that TCM compound has good curative effect with less adverse reactions in the treatment of AGA. However, the included research studies were lack in strictness and poor in quality. More high-quality randomized controlled trials are needed to furnish evidence for the efficacy of TCM. Qinpi Tongfeng Formula (QPTFF) is a TCM treatment for AGA. It has been used clinically in the First Teaching Hospital of Tianjin University of Traditional Chinese Medicine for at least 12 years. Both QPTFF combined with western medicine or bloodletting therapy have good curative effects in the treatment of gout [9, 10]. Nevertheless, rigorous randomized controlled trials have not been carried out to compare the efficacy and safety of QPTFF and NSAIDs. Thus, the purpose of this study is to evaluate the effects and safety of QPTFF in the treatment of AGA.

## 2. Materials and Methods

### 2.1. Study Design

This is a double-blind, double-dummy, multicenter, randomized, noninferiority clinical trial. The study was conducted under the *Declaration of Helsinki* and the *Good Clinical Practice Guidelines of the International Conference on Harmonization*. Meanwhile, the study followed consort (Table S1). The protocol of the study has been approved by the Ethics Committee of the First Teaching Hospital of Tianjin University of Traditional Chinese Medicine (ethics number: TYLL2021[Z] 017), and it has been registered in Chinese Clinical Trial Registry (registration number: ChiCTR2100050638).

### 2.2. Participants

Men and women aged 18–70 years were considered for enrollment if met the diagnostic criteria of AGA of the American College of Rheumatology in 2015 [11] as well as diagnostic criteria of dampness heat accumulation syndrome in the *Guidelines for the Combined Diagnosis and Treatment of Gout and Hyperuricemia* [12]. All participants signed informed consent. We conducted this clinical trial in three centers, including the First Teaching Hospital of Tianjin University of Traditional Chinese Medicine, The First Affiliated Hospital of Anhui University of Chinese Medicine, and The First Affiliated Hospital of Nanchang University.

#### 2.2.1. Inclusion Criteria

1. Patients within 72 hours of AGA attack
2. Patients with at least one attack of gout in the past
3. Patients with moderate or above arthralgia, and the visual analog scale (VAS) score for pain is ≥4
4. Patients without taking other oral traditional Chinese medicine or western medicine for AGA 72 hours before enrollment
5. Patients without taking uric-acid-lowering drugs in recent 2 weeks

#### 2.2.2. Exclusion Criteria

1. Patients diagnosed with secondary AGA caused by other diseases or drugs
2. Patients diagnosed with chronic gout
3. Patients with inflammatory arthritis such as rheumatoid arthritis, psoriatic arthritis, ankylosing spondylitis, and knee osteoarthritis
4. Patients with polyarthralgia (>4 joints)
5. Patients complicated with serious primary diseases such as cardiovascular, cerebrovascular, lung, and kidney
6. Alanine aminotransferase, aspartate aminotransferase, or serum creatinine greater than 1.5 times upper limit of normal [13]
7. Patients allergic to test drug ingredients
8. Patients currently preparing for pregnancy, being pregnant, or breastfeeding
9. Patients with active digestive ulcer or bleeding, or who have suffered or suffering from digestive ulcer or bleeding
10. Patients in other intervention studies in recent 1 month
11. Patients with mental illness or abnormal intelligence, unable to accurately express the condition or take medicine on time, and unable to finish follow-up cooperatively

#### 2.2.3. Discontinued

1. In case of intolerable adverse events, complications, or physiological changes, the researcher considered that the trial should be stopped and patients would be treated accordingly after evaluation.
2. The condition of participant did not alleviate or even got worsen within a certain period of time. Although the study was not completed, the researcher should stop the trial and take effective treatment in order to protect the participant. The curative effect of this case was determined to be invalid.
3. The participant proposed to the researcher to withdraw from the experiment voluntarily.
4. The participants who demonstrated poor compliance and were unwilling to continue participating in the study or lost to follow-up. Researchers should try to complete the last laboratory test of all withdrawn or lost cases in order to analyze their efficacy and safety. The reasons for the shedding of all shedding cases in detail and the records of the indicators that met the test requirements should be filled in the case report form (CRF). The unfinished indicators should be also filled in by the last carry forward method.

#### 2.2.4. Exclusion

During the study period, participants who used a series of combined medications at will, which will affect the assessments of curative effect, should be excluded and recorded in the CRF.

### 2.3. Methods

#### 2.3.1. Sample Size

The primary outcome measure of this study was the change in VAS score for pain from the baseline to day 8. According to the preliminary clinical trial, the mean values of VAS score changes of QPTFF and diclofenac sodium sustained-release tablets were 3.67 and 3.75, with the standard deviation of 1.22 and 0.97. For sample size estimation, PASS 15.0 software was used and noninferiority design was adopted with one-sided test selected, taking *α* = 0.025, *β* = 0.2, the ratio of the treatment group and the control group = 1 : 1, and the boundary value = −0.7. The calculated sample size for the two groups was 102 cases. Considering the potential dropout rate of about 10%, a total of 114 cases were finally included, with 57 cases in each group. According to the situation, 114 drug packaging bags were numbered, so that each center could distribute drugs according to the numbers.

#### 2.3.2. Randomization and Blinding

Excel 2013 software was used for stratified randomization for the three centers. There were 74 AGA patients in the First Teaching Hospital of Tianjin University of Traditional Chinese Medicine, 20 in the First Affiliated Hospital of Anhui University of Traditional Chinese Medicine, and 20 in the First Affiliated Hospital of Nanchang University.

The study was designed to be double-blinded and double-dummied. The treatment group used the QPTFF granule + diclofenac sodium sustained-release tablets simulant (the color, texture, taste, and smell were the same as the actual drug), while the control group used diclofenac sodium sustained-release tablets + QPTFF granule simulant (the color, texture, taste, and smell were the same as the actual drug):

1. Blinding: a trained statistician not involved with the study completed the blinding of the test medications. This study adopted a two-stage blind design. The first stage was the group code corresponding to each drug number, which was group A or group B, and the second stage was the treatment scheme adopted by group A and group B. The two-stage blind data should be placed in an opaque envelope and should not be opened during the study. All researchers who were responsible for recruiting, distributing drugs, testing indexes, and evaluating efficacy, all participants were blind to the randomization. The expert statisticians who were also blind to the randomization would conduct statistical analysis after completing the study.
2. Emergency unblinding: each drug with a number had a corresponding emergency letter, so that the patient could carry out emergency unblinding in case of serious adverse reaction events.

### 2.4. Treatment

Basic treatment: (1) avoiding cold and wet stimulation of joints, (2) no drinking, (3) low purine diet, (4) drinking more than 2000 mL water every day, and (5) prohibiting medications that affect uric acid metabolism.

#### 2.4.1. Medications

Patients in the treatment group were treated with QPTFF as granule, provided by Sichuan New Green Pharmaceutical Technology Development Company, Chengdu, China (batch no. 2107705). QPTFF has 8 components: 30 g Cortex *Fraxini* (Qin Pi), 10 g *Rhizoma Coptidis* (Huang Lian), 20 g *Semen Plantaginis* (Che Qian Zi), 30 g *Rhizome Dioscoreae Hypoglaucae* (Bi Xie), 80 g *Rhizoma Smilacis Glabrae* (Tu Fu Ling), 20 g *Radix Clematidis* (Wei Ling Xian), 30 g *Herba Siegesbeckiae* (Xi Xian Cao), and 10 g *Radix Saposhnikoviae* (Fang Feng). QPTFF was taken one bag each time, three times daily, boiled in water for each dose. The simulant of diclofenac sodium sustained-release tablets was manufactured by Tiandi Hengyi Pharmaceutical Company, Changsha, China (batch no. 201101), and was taken 0.1 g each time orally, once daily. Patients in the control group were treated with diclofenac sodium sustained-release tablets provided by Hunan Warner Pharmaceutical Company in Liuyang, China (H200677776), which was taken 0.1 g orally, once daily; QPTFF simulant (made by Sichuan New Green Pharmaceutical Technology Development Company, Chengdu, China, batch no. 2107705) was taken one bag each time, three times daily, boiled in water for each dose. The simulant was similar to the original drug in appearance, smell, and taste. All participants were treated for 7 days.

#### 2.4.2. Emergency Treatment

If the pain of participants was severe and intolerable during the study, they would be given colchicine tablets (obtained by Guangdong Pidi Pharmaceutical Company, Kaiping, China, H20113208) to assist in emergency pain relief, 0.5 mg each time, three times a day. At the same time, the medication administration would be recorded in the CRF.

### 2.5. Indicators

#### 2.5.1. Baseline Demographic Characteristics of Patients

Record the participant's name, gender, age, height, weight, body mass index (BMI), nationality, course of gout disease, allergy history, smoking history, drinking history, past medical history, and family history of gout.

#### 2.5.2. Safety Indicators

1. Vital sign: temperature, heart rate, blood pressure, and respiration were recorded on day 1 and day 8
2. Laboratory examination: routine blood test, urinalysis, liver function including alanine aminotransferase and aspartate aminotransferase, and renal function including blood urea nitrogen and serum creatinine were examined on day 1 and day 8
3. Treatment-related adverse events (TRAEs): researchers refer to the incidence of Common Terminology Criteria for Adverse Events version 5.0 [14] to record the adverse events and treatment measures in the whole process

#### 2.5.3. Primary Outcome Measure

*(1) Changes in VAS Score for Pain*. The pain degree of the participant was evaluated by VAS [15]. Researchers should record VAS score for pain before each treatment and the VAS score for final pain on day 8. A total of 8 scores were obtained.

#### 2.5.4. Secondary Efficacy Index

*(1) Joint Symptom Score*. The participant's joint tenderness, redness, swelling, and mobility were evaluated by Likert scale [16] (Table S2) at the baseline and day 8.

*(2) TCM Syndrome Score*. Researchers would evaluate the TCM syndrome score, including the main and concurrent symptoms of AGA patients (Table S3), referring to the *Guiding Principles for Clinical Research of New Traditional Chinese Medicine* [17], the quantitative integral evaluation of TCM syndrome was adopted. The higher the score, the worse the condition of AGA was. The TCM syndrome score would be recorded at the baseline on day 8.

*(3) Total Effective Rate*. The efficacy was evaluated according to the *Guiding Principles for Clinical Research of New Traditional Chinese Medicine* [18] (efficacy index = (pretreatment TCM syndrome score−posttreatment TCM syndrome score)/pretreatment TCM syndrome score × 100%) [19]. Recovery: curative effect index ≥95%; markedly effective: 70%≤ curative effect index <95%; effective: 30%≤ efficacy index <70%; ineffective: efficacy index <30%. Total effective rate = (the number of people cured + the number of people in markedly effective + the number of people in effective)/total number of people × 100%.

*(4) Pain Cure Rate*. Definition of pain cure: within 24 hours during the treatment, the VAS score for pain was 0, and the VAS score was still 0 24 hours after the treatment. The pain cure rate refers to the proportion of cured patients in the total number of patients within 7 days of treatment.

*(5) Complete Pain Relief Time*. Complete pain relief time indicated the duration of the VAS score turning to 0.

*(6) Patient Satisfaction Score*. On day 8, the participant would get an overall evaluation of the treatment, which was rated as 5 points (1 point: poor treatment effect; 2 points: slightly effective; 3 points: acceptable curative effect; 4 points: good; and 5 points: extremely good) [16].

*(7) Laboratory Index*. The levels of CRP, ESR, and serum uric acid of participants were measured at baseline on day 8.

### 2.6. Statistical Analysis

All outcome measures in this study were analyzed by the full analysis set (FAS) and per protocol set (PPS). In the FAS, for the indicators with missing data, the last observation value carry forward method was used to fill in the data. Safety indicators were analyzed by a safety set. SPSS 22.0 was used to carry out statistical analysis on the data. The quantitative data were described by mean, standard deviation, or interquartile interval *M* (P_25_, P_75_). For normal distributed data, the independent sample *t*-test or paired *t*-test was used for comparison between groups or within groups before and after the treatment, while data in skewed distribution nonparametric test should be adopted. The repeated measurement data in this study were skewed distribution, so the mixed linear model was used. The counting data were expressed by frequency and composition ratio, and the chi-square test was used for statistics; the Kaplan–Meier survival curve was used to describe the time of complete pain relief, and the log-rank test was used for comparison between groups. *P* < 0.05 indicated the difference was statistically significant. The change of VAS score was taken as the main efficacy index, and the noninferiority test was carried out according to the confidence interval method. SAS software was used to calculate the 95% confidence interval (CI) of the difference between the changes value of VAS score for pain between the treatment group and the control group before and after treatment. If the lower limit of the CI was greater than the limit value, the noninferiority was established [20]. GraphPad Prism 8 software was used to make graphics.

## 3. Results

### 3.1. Case Collection and Completion

**Figure 1:**
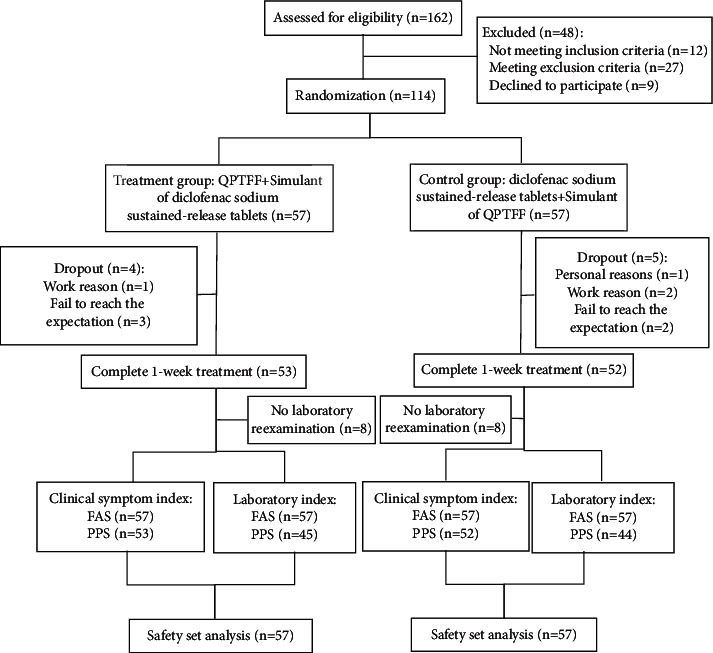
Flow diagram of AGA patients.

According to the registered protocol, 114 eligible AGA patients were included in this study from August 2021 to February 2022. In the course of the study, the clinical symptom indexes (VAS score for pain, TCM syndrome score, and joint symptom score) were measured. 105 participants completed the 7-day treatment, and 105 were finally included in the PPS, including 53 in the treatment group and 52 in the control group; 89 participants finally finished the laboratory indexes and were included in the PPS, including 45 in the treatment group and 44 in the control group (Figure 1).

### 3.2. Baseline Characteristic Analysis

There was no significant difference between the two groups in gender, age, course of gout disease, weight, height, and BMI (*P* > 0.05), as shown in Table 1. There was no significant difference in allergy history, smoking history, drinking history, past history, and family history of gout between the two groups (*P* > 0.05). There was no significant difference in temperature, heart rate, systolic blood pressure, diastolic blood pressure, and respiration between the two groups (*P* > 0.05). There was no significant difference between the two groups in VAS score for pain, joint symptom score, TCM syndrome score, ESR, CRP, and serum uric acid level (*P* > 0.05), as shown in Table 2, indicating that the two groups were comparable at baseline.

### 3.3. Clinical Efficacy

#### 3.3.1. VAS Score for Pain

**Figure 2:**
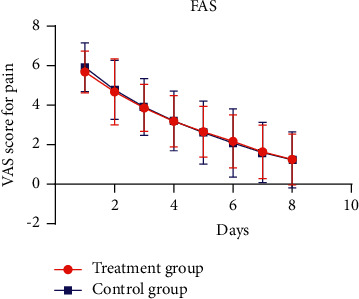
Changes in VAS score for pain between the two groups (FAS).

**Figure 3:**
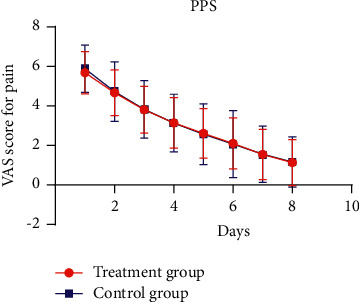
Changes in VAS scores for pain between the two groups (PPS).

The VAS score for pain of the two groups decreased gradually, and the changes are shown in Figures 2 and 3. The mixed linear model was used to compare the measurement results of VAS score at different time points between the two groups. The fixed effect analysis results of mixed linear model showed that there was no interaction effect at group ^*∗*^ time point (*P* > 0.05), and the overall curative effect difference between the two groups was not statistically significant (*P* > 0.05), as shown in Table 3. There was no significant difference in VAS score between the two groups at different time points (*P* > 0.05), as shown in Table 4. In the FAS, the 95% CI of the difference between the eighth time and baseline VAS scores of the two groups was (−0.57, 0.42), and its lower limit was greater than the boundary value of −0.7, so the noninferiority was established. In the PPS set, the 95% CI of the difference between the eighth time and the baseline VAS scores the two groups was (−0.48, 0.47), and its lower limit was greater than the boundary value of −0.7, so the noninferiority was established. In conclusion, the noninferiority test of this study is qualified.

#### 3.3.2. Joint Symptom Score

The joint symptom scores of the two groups after the treatment were better than those before the treatment (FAS and PPS, *P* < 0.05). After the treatment, there was no significant difference in the joint symptom scores between the treatment group and the control group (*P* > 0.05), as shown in Table 5.

#### 3.3.3. TCM Syndrome Score

In FAS and PPS, the TCM syndrome scores of the two groups after the treatment were better than those before the treatment and the difference was statistically significant (*P* < 0.05). After the treatment, there was no statistically significant difference between the TCM syndrome scores of the treatment group and the control group (*P* > 0.05), as shown in Table 6.

#### 3.3.4. Total Effective Rate

In FAS, the total effective rate was 89.47% in the treatment group and 87.72% in the control group, and there was no significant difference between the two groups (*P* > 0.05). In PPS, the total effective rate was 96.23% in the treatment group and 96.15% in the control group, and there was no significant difference between the two groups (*P* > 0.05), as shown in Table 7.

#### 3.3.5. Pain Cure Rate

In FAS, the pain cure rate was 35.09% in the treatment group and 38.60% in the control group, and there was no significant difference between the two groups (*P* > 0.05). In PPS, the pain cure rate was 35.84% in the treatment group and 38.46% in the control group, and there was no significant difference between the two groups (*P* > 0.05), as shown in Table 8.

#### 3.3.6. Comparison of Complete Pain Relief Time between the Two Groups

**Figure 4:**
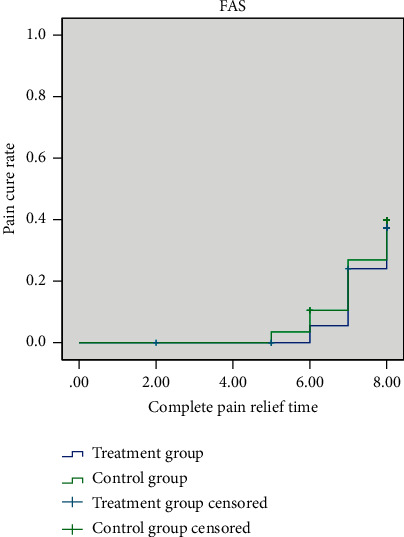
Survival curves for complete pain relief time (FAS).

**Figure 5:**
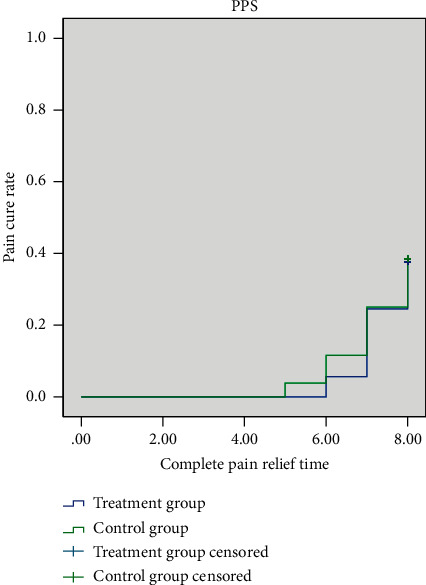
Survival curves for complete pain relief time (PPS).

In FAS, the median time of complete pain relief time in both the treatment group and the control group was 7.65 days (Figure 4). Using the log-rank test, there was no significant difference between the two groups (*P*=0.701). In PPS, the median time of complete pain relief time in the treatment group and the control group was 7.65 days. The survival curve of the two groups is shown in Figure 5. Using the log-rank test, there was no significant difference between the two groups (*P*=0.85).

#### 3.3.7. Patient Satisfaction Score

The patient satisfaction score adopted PPS. The results showed that there was no statistical difference between the two groups (*P* > 0.05) (see Table 9).

#### 3.3.8. Laboratory Index

In FAS and PPS, the levels of ESR and CRP in the two groups after the treatment were lower than those before the treatment and the differences within the two groups were statistically significant (*P* < 0.001). After the treatment, there was no statistically significant difference in the levels of ESR and CRP between the treatment group and the control group (*P* > 0.05), as shown in Tables 10 and 11. In FAS and PPS, the level of serum uric acid in the treatment group after the treatment was significantly lower than that before the treatment (*P* < 0.001), and there was no significant difference in the level of serum uric acid in the control group before and after the treatment (*P* > 0.05). After the treatment, the level of serum uric acid in the treatment group was significantly lower than that in the control group (*P* < 0.05), as shown in Table 12.

### 3.4. Safety Evaluation

During the treatment, routine blood test and urinalysis did not show abnormalities in the two groups. Only 23 patients had elevated leukocytes stimulated by inflammation before the treatment, but they all returned to normal level after the treatment. Another 9 patients had elevated platelet count before the treatment, but decreased after the treatment. There was no abnormal increase or decrease in vital signs in the two groups after the treatment. The TRAEs were 7.02% in the treatment group and 26.32% in the control group. The TRAEs of the treatment group were significantly lower than those of the control group (*P* < 0.05), as shown in Table 13.

## 4. Discussion

AGA is caused by the deposition of urate crystals in the articular cavity, manifested as redness, swelling, and severe pain of the joints [21]. International guidelines recommend NSAIDs as the first-line treatment of AGA [22, 23], and diclofenac sodium sustained-release tablets are commonly used as positive controlled medicines in the treatment of AGA [24–26]. Therefore, diclofenac sodium sustained-release tablet was chosen as the controlled medicine in this study. VAS score for pain is the most widely used tool to measure pain intensity in clinic [27, 28], which is often used to evaluate the condition changes in joint pain of AGA [29]. In addition, the joint symptom score scale in this study includes the condition of joint tenderness, joint redness, joint swelling, and joint activity, which can comprehensively reflect the clinical manifestations of affected joints. Meanwhile, according to the requirements of the *Guiding Principles for Clinical Research of New Traditional Chinese Medicine* [17], the TCM syndrome score scale is used to evaluate the TCM syndrome of patients, which is widely used in the efficacy evaluation of TCM therapy [9, 19]. ESR and CRP are commonly used as inflammatory indicators in clinic and play an important guiding role in judging the progress of inflammation [30]. Serum uric acid level is not only a diagnostic indicator of AGA but also an indicator of its prognosis. The level of serum uric acid is closely related to the recurrence rate of gouty arthritis [31], so we also listed serum uric acid as the outcome measure. Nevertheless, the main aim of the treatment to AGA is to control inflammation and relieve pain, and serum uric acid was set as the secondary outcome measure.

In this study, the improvement of patients' pain is the primary outcome measure of the study. The VAS score for pain of participants on day 8 was significantly lower than that at baseline, indicating that both treatments could effectively reduce the pain of patients. The noninferiority test proved that the lower confidence interval of the difference value of VAS score for pain (from baseline to day 8) between the two groups is greater than the lower limit (FAS and PPS, lower limit >−0.7). There was no significant difference in complete pain relief time and pain cure rate between the two groups, which suggested that the analgesic effect of QPTFF was not inferior to that of diclofenac sodium sustained-release tablets. The results of secondary outcome measures showed that both treatments had a great improvement in the joint symptom score, TCM syndrome score, ESR, and CRP, but without significant difference, indicating that QPTFF and diclofenac sodium sustained-release tablets could significantly improve the patients' symptoms and inflammatory indicators with equal effects. However, QPTFF had more advantages than diclofenac sodium sustained-release tablets in reducing the serum uric acid level. After the treatment, the satisfaction survey was carried out on the patients, and the results showed that the subjective feeling of the patients in the two groups was equal.

In terms of safety evaluation, the rate of TRAEs in the treatment group was significantly lower than that in the control group and the patients in the treatment group had no liver and kidney function injury, but discomfort in the stomach. To timely monitor the changes of liver and renal function, we set the abnormal liver and renal function as higher than the upper limit of the normal, or abnormal liver and renal function before treatment, while further increased after treatment to protect the patient's health. Although the reported percentage of abnormal liver and renal function was higher in the control group, there was no serious liver and renal injury occurred in the patients. The side effects of nonsteroidal drugs on the digestive system have been widely concerned by clinical workers. Clinical studies have shown that they will increase the risk of digestive tract, cardiovascular, and kidney disease [32]. Although the treatment course of this study was short, it has been observed that patients in the control group had abnormal liver and kidney function and adverse reactions of digestive tract. Moreover, clinical studies have pointed out that long-term use of diclofenac sodium could increase the risk of upper digestive tract, especially in elderly patients [33]. Our study suggests that QPTFF is safer than diclofenac sodium sustained-release tablets.

QPTFF is optimized and improved from Qinpi powder in the ancient Chinese book *Taiping Shenghui recipe* (AD 992). This compound contains *Cortex Fraxini* (Qin Pi), *Rhizoma Coptidis* (Huang Lian), *Semen Plantaginis* (Che Qian Zi), *Rhizome Dioscoreae Hypoglaucae* (Bi Xie), *Rhizoma Smilacis Glabrae* (Tu Fu Ling), *Radix Clematidis* (Wei Ling Xian), *Herba Siegesbeckiae* (Xi Xian Cao), and *Radix Saposhnikoviae* (Fang Feng). QPTFF has the functions of clearing heat and detoxification, removing dampness and turbidity, dredging arthralgia, and relieving pain. The total coumarin of Qinpi in *Cortex Fraxini* can reduce uric acid by inhibiting the activity of xanthine oxidase [34]. *Cortex Fraxini* extract can reduce the level of urate anion transporter 1 (URAT1), so it has the effect of reducing uric acid [35]. Aesculetin B and Aesculetin A can also inhibit the release of inflammatory factors [36]. The components in *Rhizoma Smilacis Glabrae*, such as colchicine, syringic acid, and catechin, can inhibit the expression of inflammatory factors and have strong anti-inflammatory effects [37]. *Rhizoma Smilacis Glabrae* can reduce the serum uric acid concentration of mouse hyperuricemia model by inhibiting xanthine oxidase activity [38]. Berberine is an important component of *Rhizoma Coptidis*, which could inhibit the activation of NLRP3 inflammatory bodies and prevent IL-1*β* to resist inflammation [39]. Berberine in *Rhizoma Coptidis* can reduce the serum uric acid level and protect renal function by inhibiting the activation of NLRP3 inflammatory bodies and the abnormal expression of URAT1 [40]. There are sesquiterpenoids, diterpenoids, flavonoids, and other compounds in *Herba Siegesbeckiae*, which have anti-inflammatory and analgesic effects [41]. The extract of *Herba Siegesbeckiae* can also inhibit the activity of xanthine oxidase and reduce serum uric acid [42]. *Rhizome Dioscoreae Hypoglaucae* has anti-inflammatory and analgesic effects [43, 44]. The extract of *Rhizome Dioscoreae Hypoglaucae* can promote the excretion of uric acid by regulating the levels of organic anion transporter 1, murat1, and organic cation transporter 2 and has the effect of reducing uric acid [45]. There are many components with xanthine oxidase inhibitor effect in *Semen Plantaginis*, such as luteolin, mullein glycoside, golden sage grass flavin, so it has the effect of reducing uric acid [46]. *Plantain polysaccharide* may have renal protective effect by downregulating the expression of NLRP3, ASC, and caspase-1 protein and inhibiting the release of downstream inflammatory factors [47]. *Radix Clematidis* inhibits NF-*κ*B and MAPK pathways in macrophages to reduce the production of proinflammatory factors, to function in anti-inflammatory and analgesic [48]. Polysaccharide of *Radix Saposhnikoviae* has anti-inflammatory and analgesic effects by regulating the expression of p53 and inhibiting the release of inflammatory factors [49]. The extract of *Radix Saposhnikoviae* can reduce blood uric acid by inhibiting the activity of xanthine oxidase [50]. Therefore, these drugs can exert the clinical efficacy of anti-inflammatory and analgesic, reduce serum uric acid, and improve the symptoms of gout patients.

However, our study still has some limitations. This study only uses common efficacy indicators for observation, without the observation of the changes of immune indicators and images in AGA. Although the sample size of this study was estimated by the PASS 15.0 software, it was only the minimum sample size required by clinical practice, and there were only 3 hospitals in the study. In the future, more high-quality multicenter, large-sample randomized controlled trials should be carried out to observe the changes of immune indicators and images with AGA.

## 5. Conclusions

QPTFF can improve the symptoms and signs of patients with AGA, as well as the inflammatory indexes and serum uric acid level. Its analgesic effect is not inferior to diclofenac sodium sustained-release tablets, but it has more advantages in reducing the serum uric acid level and the rate of treatment-related adverse events. Therefore, QPTFF is an effective clinical treatment for AGA.

## Figures and Tables

**Table 1:** Basic characteristics (FAS) of AGA patients ($\bar{x}$ ± *s*/*M* (*P*_25_, *P*_75_)/*n* (%)).

	Characteristics		Treatment group	Control group	*P* value
FAS	Gender	Male	56 (98.2%)	56 (98.2%)	1.000
		Female	1 (1.80%)	1 (1.80%)	
	Age (years)		41.68 ± 11.51	31.50 (26, 52)	0.247
	Course of disease (months)		50.00 (15.50, 77)	30.5 (0.25, 61.75)	0.852
	Weight (kg)		86.65 ± 15.80	82.56 ± 16.82	0.184
	Height (cm)		175.00 ± 6.07	173.47 ± 6.30	0.190
	BMI (kg/m^2^)		28.24 ± 4.74	27.29 ± 4.54	0.275

PPS	Gender	Male	52 (98.11%)	51 (98.08%)	1.000
		Female	1 (1.89%)	1 (1.89%)	
	Age (years)		41 ± 11.65	29.5 (25.75, 48.25)	0.487
	Course of disease (months)		59.50 (17.50, 89.00)	26 (0.00, 61.25)	0.430
	Weight (kg)		86.58 ± 15.74	83.00 ± 17.42	0.272
	Height (cm)		176.02 ± 6.17	173.62 ± 6.20	0.248
	BMI (kg/m^2^)		28.21 ± 4.73	27.36 ± 4.62	0.353

**Table 2:** Baseline comparison of clinical efficacy indicators ($\bar{x}$ ± *s*/*M* (*P*_25_, *P*_75_)).

	Characteristics	Treatment group	Control group	*P* value
FAS	VAS score for the pain	6.00 (5.00, 6.00)	5.00 (5.00, 6.00)	0.292
	Joint symptom score	7.00 (5.00, 7.50)	7.00 (7.00, 8.00)	0.371
	TCM syndrome score	26.73 ± 5.26	27.26 ± 4.46	0.566
	ESR (mm/h)	24.79 ± 14.49	22.95 ± 13.48	0.484
	CRP (mg/L)	40.18 ± 24.36	25.08 (11.66, 49.45)	0.854
	Serum uric acid (*μ*mol/L)	565.97 ± 129.96	572.01 ± 119.20	0.796

PPS	VAS score for the pain	5.50 (5.00, 6.75)	5.00 (4.75, 6.00)	0.346
	Joint symptom score	7.00 (5.25, 7.75)	7.00 (7.00, 8.00)	0.377
	TCM syndrome score	26.75 ± 5.39	27.29 ± 4.60	0.587
	ESR (mm/h)	23.31 ± 13.24	22.50 (13, 30.75)	0.912
	CRP (mg/L)	15.93 (7.28, 44.25)	18.07 (11.54, 51.58)	0.453
	Serum uric acid (*μ*mol/L)	565.39 ± 136.39	577.51 ± 120.78	0.659

**Table 3:** Analysis of mixed linear models of VAS scores for pain between the two groups.

	Characteristics	Statistics (*F*)	*P* value
FAS	Group	0.091	0.762
	Time point	148.619	<0.001
	Group ^*∗*^ time point	0.148	0.994

PPS	Group	0.087	0.768
	Time point	148.950	<0.001
	Group ^*∗*^ time point	0.092	0.999

**Table 4:** Comparison of VAS scores for pain at each time point between the two groups.

	Characteristics	Group (*I*)	Group (*J*)	Mean difference (*I*−*J*)	*P* value
FAS	Baseline	Treatment group	Control group	0.02	0.946
	2^nd^	Treatment group	Control group	0.04	0.887
	3^rd^	Treatment group	Control group	0.07	0.781
	4^th^	Treatment group	Control group	0.05	0.855
	5^th^	Treatment group	Control group	−0.01	0.968
	6^th^	Treatment group	Control group	−0.05	0.839
	7^th^	Treatment group	Control group	−010	0.694
	8^th^	Treatment group	Control group	−0.23	0.374

PPS	Baseline	Treatment group	Control group	−0.20	0.939
	2^nd^	Treatment group	Control group	−0.01	0.980
	3^rd^	Treatment group	Control group	0.40	0.877
	4^th^	Treatment group	Control group	0.40	0.871
	5^th^	Treatment group	Control group	0.01	0.973
	6^th^	Treatment group	Control group	−0.02	0.936
	7^th^	Treatment group	Control group	−0.06	0.827
	8^th^	Treatment group	Control group	−0.21	0.432

**Table 5:** Comparison of joint symptom scores between the two groups (*M* (*P*_25_, *P*_75_)).

	Group	*N*	Before treatment	After treatment	Comparison between groups
					*P* value
FAS	Treatment group	57	6.00 (7.00, 8.00)	0.00 (0.00, 1.00)	<0.001
	Control group	57	6.00 (7.00, 8.00)	0.00 (0.00, 0.00)	<0.001
	Comparison between groups	*P* value		0.542	

PPS	Treatment group	53	7.00 (5.25, 7.75)	0.00 (0.00, 1.00)	<0.001
	Control group	52	7.00 (7.00, 8.00)	0.00 (0.00, 0.00)	<0.001
	Comparison between groups	*P* value		0.397	

**Table 6:** Comparison of TCM syndrome scores between the two groups ($\bar{x}$ ± *s*/*M* (*P*_25_, *P*_75_)).

	Group	*N*	Before treatment	After treatment	Comparison between groups
					*P* value
FAS	Treatment group	57	26.74 ± 5.26	3 (2, 5)	<0.001
	Control group	57	27.26 ± 4.46	10.21 ± 7.54	<0.001
	Comparison between groups	*P* value		0.325	

PPS	Treatment group	53	26.75 ± 5.39	2.5 (2, 4)	<0.001
	Control group	52	27.29 ± 4.60	8.60 ± 5.60	<0.001
	Comparison between groups	*P* value		0.329	

**Table 7:** Comparison of total effective rate between the two groups (*n* (%)).

Curative effect index	FAS		PPS	
	Treatment group (*n* = 57)	Control group (*n* = 57)	Treatment group (*n* = 53)	Control group (*n* = 52)
Cured	3 (5.26%)	2 (3.51%)	3 (5.66%)	2 (3.85%)
Markedly effective	28 (49.12%)	26 (45.61%)	28 (52.83%)	26 (50.00%)
Effective	20 (35.09%)	22 (38.60%)	20 (37.74%)	22 (42.31%)
Ineffective	6 (10.53%)	7 (12.28%)	2 (3.77%)	2 (3.85%)
Total effective rate	89.47%	87.72%	96.23%	96.15%
*P* value	0.789		0.809	

**Table 8:** Comparison of pain cure rate between the two groups (*n* (%)).

	Group	*N*	Pain cure rate	*P* value
FAS	Treatment group	57	20 (35.09%)	0.698
	Control group	57	22 (38.60%)	

PPS	Treatment group	53	19 (35.84%)	0.597
	Control group	52	20 (38.46%)	

**Table 9:** Patient satisfaction score between the two groups (*M* (*P*_25_, *P*_75_)).

	Group	*N*	Score	Statistics	*P* value
PPS	Treatment group	53	4 (4, 4)	*Z* = −1.850	0.064
	Control group	52	5 (4, 5)		

**Table 10:** Comparison of ESR levels between the two groups ($\bar{x}$ ± *s*/*M* (*P*_25_, *P*_75_)).

	Group	*N*	Before treatment	After treatment	Comparison between groups
					*P* value
FAS	Treatment group	57	24.79 ± 14.49	8 (5, 16.5)	<0.001
	Control group	57	22.95 ± 13.48	14 (4.5, 23)	<0.001
	Comparison between groups	*P* value		0.656	

PPS	Treatment group	45	23 (12, 35)	5.5 (3, 11.5)	<0.001
	Control group	44	22.50 (13, 30.75)	5.5 (3, 13.5)	<0.001
	Comparison between groups	*P* value		0.297	

**Table 11:** Comparison of CRP levels between the two groups ($\bar{x}$ ± *s*/*M* (*P*_25_, *P*_75_)).

	Group	*N*	Before treatment	After treatment	Comparison between groups
					*P* value
FAS	Treatment group	57	40.18 ± 24.36	5.37 (3.13, 8.45)	<0.001
	Control group	57	25 (10.99, 50.41)	5.39 (3.13, 16.00)	<0.001
	Comparison between groups	*P* value		0.353	

PPS	Treatment group	45	15.93 (7.28, 44.25)	3.13 (3.13, 4.42)	<0.001
	Control group	44	18.07 (11.54, 51.58)	3.13 (3.13, 4.05)	<0.001
	Comparison between groups	*P* value		0.309	

**Table 12:** Comparison of serum uric acid levels between the two groups ($\bar{x}$ ± *s*).

	Group	*N*	Treatment group	Control group	Comparison between groups
					*P* value
FAS	Treatment group	45	565.97 ± 126.96	523.33 ± 100.64	0.004
	Control group	44	572.01 ± 119.20	562.20 ± 101.68	0.419
	Comparison between groups	*P* value		0.043	

PPS	Treatment group	45	565.39 ± 136.39	511.38 ± 96.47	0.003
	Control group	44	577.51 ± 120.78	564.80 ± 98.25	0.420
	Comparison between groups	*P* value		0.011	

**Table 13:** Comparison of adverse reactions between the two groups (*n* (%)).

The treatment-related adverse events	Treatment group (*n* = 57)		Control group (*n* = 57)	
	*N*	Adverse reaction classification	*N*	Adverse reaction classification
Abnormal liver function	0 (0%)	No	4 (7.02%)	Grade 2
Abnormal renal function	0 (0%)	No	2 (3.51%)	Grade 1
Nausea	0 (0%)	No	2 (3.51%)	Grade 1
Vomit	2 (3.51%)	Grade 1	0 (0%)	No
Acid reflux	0 (0%)	No	1 (1.75%)	Grade 1
Diarrhea	2 (3.51%)	Grade 1	3 (5.26%)	Grade 1
Stomachache	0 (0%)	No	2 (3.51%)	Grade 2
Dizzy	0 (0%)	No	1 (1.75%)	Grade 1
Total	4 (7.02%)		15 (26.32%)	
*P* value	0.012			

## Data Availability

The datasets used and analyzed in the current study are available from the corresponding author on reasonable request (Wei Liu: fengshiliuwei@163.com).

## Abbreviations

TCM: Traditional Chinese medicine
AGA: Acute gouty arthritis
QPTFF: Qinpi Tongfeng Formula
VAS: Visual analog scale
ESR: Erythrocyte sedimentation rate
CRP: C-reactive protein
TRAEs: Treatment-related adverse events
FAS: Full analysis set
PPS: Per protocol set
NSAIDs: Nonsteroidal anti-inflammatory drugs
CRF: Case report form
BMI: Body mass index
CI: Confidence interval.

## Supplementary Materials

Supplementary Materials Table S1: the CONSORT 2010 checklist. Table S2: joint symptom score. Table S3: TCM syndrome score.
