# Supplementary material for: Efficacy and Safety of Qinpi Tongfeng Formula in the Treatment of Acute Gouty Arthritis: A Double-Blind, Double-Dummy, Multicenter, Randomized Controlled Trial
Source: Evid Based Complement Alternat Med. 2022 Jul 12;2022:7873426. doi: 10.1155/2022/7873426 (PMC9296295; doi:10.1155/2022/7873426)
Supplement: Supplementary Materials — Table S1: the CONSORT 2010 checklist. Table S2: joint symptom score. Table S3: TCM syndrome score. [file 7873426.f1.zip › 7873426.f1/Table S2 Joint symptom score (3).docx]

Table S2 Joint symptom score

| Likert scale score method | | Scores |
| --- | --- | --- |
| Joint tenderness | 0=No pain; 1= Patient states ‘there is pain; 2=Patient states ‘there is pain’ and withdraws |  |
| Joint erythema | 0=Absent; 1=Not assessable; 2= Present |  |
| Joint swelling | 0=No swelling; 1=Palpable; 2=Visible; 3=Bulging beyond joint margins |  |
| Joint activity | 0=No restricted; 1= Moderate restricted; 2=Significantly restricted; 3=Unbearable, cannot take care of themselves |  |
