# Supplementary material for: Efficacy and Safety of Qinpi Tongfeng Formula in the Treatment of Acute Gouty Arthritis: A Double-Blind, Double-Dummy, Multicenter, Randomized Controlled Trial
Source: Evid Based Complement Alternat Med. 2022 Jul 12;2022:7873426. doi: 10.1155/2022/7873426 (PMC9296295; doi:10.1155/2022/7873426)
Supplement: Supplementary Materials — Table S1: the CONSORT 2010 checklist. Table S2: joint symptom score. Table S3: TCM syndrome score. [file 7873426.f1.zip › 7873426.f1/Table S3 TCM syndrome score (3).docx]

**Table S3 TCM syndrome score**

| **TCM syndrome score** | | | | | |
| --- | --- | --- | --- | --- | --- |
| **Main symptom** | **0 points** | **2 points** | **4 points** | **6 points** | **Score** |
| **Joint pain** | Grade 0: normal, no pain | Grade 1: mild, tolerable, but not affecting work and life | Grade 2: moderate, affecting work but not life | Grade 3: severe, affecting life and sleep |  |
| **Joint swelling** | Grade 0: normal, no swelling | Grade 1: mild, mild swelling, no obvious change of skin texture and obvious bony signs | Grade 2: moderate, moderate swelling, obvious change or even disappearance of skin texture | Grade 3: severe, severe swelling, tight skin, disappearance of skin texture and bony signs |  |
| **Joint tenderness** | Grade 0: normal, no tenderness | Grade 1: mild, mild tenderness | Grade 2: moderate, moderate tenderness, tolerable, frowning discomfort | Grade 3: severe, severe tenderness, untouchable pain |  |
| **Joint skin temperature** | Grade 0: normal, no joint fever | Grade 1: mild, local joint contact heat, no conscious fever | Grade 2: moderate, local joint contact heat, accompanied by conscious heat | Grade 3: severe, local joint contact heat, with conscious burning |  |
| Joint skin color | Grade 0: normal, no joint skin color change | Grade 1: mild, slightly red complexion | Grade 2: moderate, red complexion | Grade 3: severe, dark red complexion |  |
| Joint function | Grade 0: normal, no activity restriction | Grade 1: mild, mild activity restriction | Grade 2: moderate, unable to engage in general activities, but self-care | Grade 3: severe, unbearable pain during activities, bedridden, unable to take care of themselves |  |
| **Concurrent disease** | **0 points** | **2 points** | **4 points** | **6 points** | **Score** |
| Fever | Grade 0: normal, no fever | Grade 1: mild, 37.3 ℃ ≤ fever ＜ 38 ℃ | Grade 2: moderate, 38 ℃ ≤ fever ＜ 39 ℃ | Grade 3: severe, high fever, fever ≥ 39 ℃ |  |
| Thirsty | Grade 0: normal, no thirst | Grade 1: mild, without drinking water | Grade 2: moderate, drinking water is required when necessary | Grade 3: severe, frequent drinking, no improvement in drinking thirst |  |
| Fidgety | Grade 0: normal, without irritability | Grade 1: mild, occasionally irritable | Grade 2: moderate, irritability is more frequent, but it does not affect sleep | Grade 3: severe, irritable, affecting sleep |  |
| Limb sleepiness | Grade 0: normal, no limb sleepiness | Grade 1: mild, slightly sleepy limbs, heavy without affecting activities | Grade 2: moderate, heavy limb sleepiness and reduced activity | Grade 3: severe, obvious sleepiness, no desire to move |  |
| Yellow urine | Grade 0: normal, urine color is normal | Grade 1: mild, slightly yellow urine | Grade 2: moderate, short yellow urine | Grade 3: severe, short urination, dark yellow |  |
| Stool viscosity | Grade 0: normal, normal stool | Grade 1: mild, slightly sticky or occasionally sticky stool | Grade 2: moderate, stool sticky or often sticky | Grade 3: severe, stool is obviously sticky or always sticky |  |
